# Network Pharmacology and Bioinformatics Approach Reveals the Multi-Target Pharmacological Mechanism of *Fumaria indica* in the Treatment of Liver Cancer

**DOI:** 10.3390/ph15060654

**Published:** 2022-05-25

**Authors:** Sara Batool, Muhammad Rizwan Javed, Sidra Aslam, Fatima Noor, Hafiz Muhammad Faizan Javed, Riffat Seemab, Abdur Rehman, Muhammad Farhan Aslam, Bilal Ahamad Paray, Aneela Gulnaz

**Affiliations:** 1 Department of Bioinformatics and Biotechnology, Government College University Faisalabad (GCUF), Allama Iqbal Road, Faisalabad 38000, Pakistan; sara93batool@gmail.com (S.B.); faitmanoor23@gcuf.edu.pk (F.N.); riffatseemab1@gmail.com (R.S.); abdurrehman93@gcuf.edu.pk (A.R.); 2 Allied Hospital, Faisalabad 38000, Pakistan; mfaizanjaved@gmail.com; 3 School of Biological Sciences, University of Edinburgh, Edinburgh P.O. Box EH9 3FF, UK; m.f.aslam@sms.ed.ac.uk; 4 Department of Zoology, College of Science, King Saud University, P.O. Box 2455, Riyadh 11451, Saudi Arabia; bparay@ksu.edu.sa; 5 College of Pharmacy, Woosuk University, Wanju-gun 55338, Korea; draneela@woosuk.ac.kr

**Keywords:** network pharmacology, *Fumaria indica*, liver cancer, active constituents, molecular docking

## Abstract

Liver cancer (LC), a frequently occurring cancer, has become the fourth leading cause of cancer mortality. The small number of reported data and diverse spectra of pathophysiological mechanisms of liver cancer make it a challenging task and a serious economic burden in health care management. *Fumaria indica* is a herbaceous annual plant used in various regions of Asia to treat a variety of ailments, including liver cancer. Several in vitro investigations have revealed the effectiveness of *F. indica* in the treatment of liver cancer; however, the exact molecular mechanism is still unrevealed. In this study, the network pharmacology technique was utilized to characterize the mechanism of *F. indica* on liver cancer. Furthermore, we analyzed the active ingredient-target-pathway network and uncovered that Fumaridine, Lastourvilline, N-feruloyl tyramine, and Cryptopine conclusively contributed to the development of liver cancer by affecting the MTOR, MAPK3, PIK3R1, and EGFR gene. Afterward, molecular docking was used to verify the effective activity of the active ingredients against the prospective targets. The results of molecular docking predicted that several key targets of liver cancer (along with MTOR, EGFR, MAPK3, and PIK3R1) bind stably with the corresponding active ingredient of *F. indica*. We concluded through network pharmacology methods that multiple biological processes and signaling pathways involved in *F. indica* exerted a preventing effect in the treatment of liver cancer. The molecular docking results also provide us with sound direction for further experiments. In the framework of this study, network pharmacology integrated with docking analysis revealed that *F. indica* exerted a promising preventive effect on liver cancer by acting on liver cancer-associated signaling pathways. This enables us to understand the biological mechanism of the anti liver cancer activity of *F. indica*.

## 1. Introduction

Liver cancer is the fourth major cause of cancer-related deaths globally. According to a report from Cancer Research UK, liver cancer will be one of the fastest-growing malignancies by 2035 [1]. It is a fatal condition that affects people with hepatitis A or C, fatty liver disease, and diabetes, and it has been associated with excessive alcohol consumption, smoking, and dietary toxins [2]. Some hereditary variables may also play a role in the development of this disease. Abnormalities in the cell cycle, metabolic networks, and inflammatory response may also lead towards liver cancer [3]. Despite well-known threats for liver cancer such as viral hepatitis and metabolic syndrome, liver cancer is detected late in the majority of cases [4]. Despite substantial advances in liver cancer treatment over the last few years, the majority of drugs still fail to produce satisfying results in patients.

*Fumaria indica* (synonym: *Fumaria parviflora*, Fumariaceae) is a wild plant that has been used in traditional ayurvedic texts to treat a wide range of illnesses, including skin diseases, tropical diseases, cardiovascular disorder, circulatory disease, fever, and headache, as well as many other things [5]. The latest pharmacological studies suggest that *F. indica* possesses anticancer, antidiabetic, antimicrobial, and antiinflammatory properties due to the presence of natural bioactive compounds [6]. The Fumitory genus is distributed in central Asia. Previous studies indicated that *F. indica* is a fundamental and a unique source of a variety of potential phytochemicals, making it a useful and versatile plant with a wide range of medicinal properties [7]. Hussain et al. [8] evaluated the chemo-preventive effect of *F. indica* against N-nitrosodiethylamine and CCl4-induced hepatocellular carcinoma in Wistar rats. Their findings powerfully support that treatment with *F. indica* significantly reduced the liver injury and restored the entire liver cancer markers

Network pharmacology (NP) is a field of study that is based on systems biology and multi-directional pharmacology [9]. Rather than focusing on the single-target procedure in which drugs work, it looks at the multi-target process in which drugs work [10]. Hence, the new drug development process and strategy combines computer science, molecular biology, and pharmacology [11,12,13,14,15]. A systematic and comprehensive exposition of ‘disease–target protein–drug’ linkages may be constructed utilizing professional networks and existing resources such as genes, proteins, illnesses, and medications [16]. Based on the component-target network, this study will also be conducted more in-depth after it has been set up. Molecular docking is a computer programme that simulates how ligands and receptors might interact in real life. It also predicts how the ligands and receptors might interact. Molecular docking is used to learn more about how ligands and receptors work together, as well as how to make new drugs. The potential of using bioactive constituents to reform medicine in the future is thrilling, and chances for healing a variety of disorders are encouraging. Recently Yu et al. [17] implemented a network pharmacology-based approach to examine the active components of Gupi Xiaoji prescription for the treatment of liver cancer. Therefore, network pharmacology apportioned a strong technique for finding bioactive ingredients and the mechanism of action for traditional medicine formula used to treat illness and diseases [18].

In the current study, a comprehensive NP-based approach was used to explore the active phytochemicals of *F. indica.* To the best of our knowledge, this is the first study to combine bioinformatics analysis with NP to reveal the mechanism of *F. indica* for liver cancer treatment. This research study will add to our understanding of the molecular mechanism of *F. indica*’s anti-liver effect and help to speed up the drug discovery process. The bioactive components and putative molecular network mechanism of *F. indica* against liver cancer are examined from a systematic and molecular level using network pharmacology along with molecular docking analysis. In the near future, wet lab research is also proposed to explore the additional pharmacological potentials.

## 2. Results

### 2.1. Screening of Active Compounds and Targets

A total of 62 active components of *Fumaria indica* were found in the literature. Out of these, 15 (Protopine, Fumaridine, Parfumine, Lastourvilline, N-feruloyl tyramine, Fumarizine, Paprarine, Cryptopine, Berberine, Stigmesterol, Campesterol, Papaverine, Oxyhydrastinine, Noscapine, Apigenin) components met the screening standards of the DL index ≥ 0.18 and OB ≥ 30%. Furthermore, from the SwissTargetPrediction database, we found 1500 genes that could be the target of the 15 active ingredients (Table 1). After finding the most promising targets for drugs, a total of 38,477 genes linked to liver cancer were found in the GeneCards and DisGeNET databases. Later, a Venn diagram was used to figure out the common targets of both liver cancer and compound linked genes. A total of 557 genes from *F. indica* that could fight against liver cancer were considered as key targets.

### 2.2. Compounds-Target Network Construction

A total of 15 active compounds from *F. indica* were found to be satisfactory. To construct the ‘active compound–targeted genes–connected pathway’ network diagram, 15 active compounds, 1500 key targets, and their associated pathways with high gene count were chosen. Each of these active compounds have multiple targets, which shows that many targets induce a synergistic effect when *F. indica* is used as an anti-hepatic cancer agent. The degree of these 12 compounds in the compound-targeted genes-connected pathways network was then assessed (Table 2). As indicated in Table 2, alkaloids together with tyramines have comparably maximum degree; however, the degree of both alkylamides and steroids are comparatively low as compared to alkaloids and tyramines. Furthermore, from these 12 compounds, 4 compounds were selected for docking analysis: two alkaloids with a higher degree of connectivity, especially Fumaridine and Cryptopine, one tyramine compound, namely N-Feruloyltyramine, and one alkylamides compound, namely Lastourvilline.

### 2.3. PPI Network Construction

**Figure 1:**
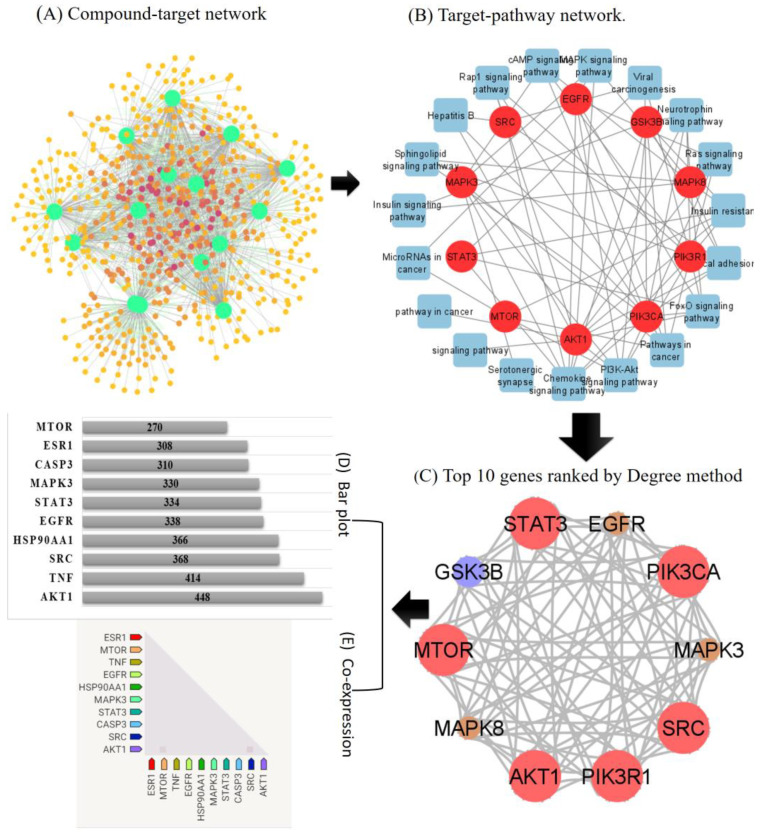
Network pharmacology-based analysis of multi-compound, multi-target, and multi-pathway treatment for liver cancer. (**A**) Network diagram of compounds and their targets. Size and color of gene and compound’s nodes represent their degree (**B**) Network diagram of target genes–enrichment pathways. The blue square indicates the pathways and pink nodes indicates the target. (**C**) Top 10 genes ranked by degree method. (**D**) The bar plot of the PPI network. (**E**) Observed expression of 10 target genes in *Homo sapiens*.

The 557 genes that overlapped were uploaded to the STRING database to build a PPI network. A PPI network shows how different targets work together during the development of a disease. The nodes and their connections show how these targets work together (Figure 1A). Later, Cytoscape was utilized at the PPI network of genes that overlapped (Figure 1C). AKT1 (224), TNF (207), SRC (184), EGFR (169), STAT3 (169), MAPK3 (165), CASP3 (MTOR), MAPK1 (134), EP300 (115), PIK3CA (109), CDC42 (107), MDM2 (104), MAPK14 (95), PIK3R1 (94), MAPK8 (77), and GSK3B (75) showed the highest degree (Figure 1D). This means that the highest degree genes are greatly linked to each other; thus, all of these genes might be hub targets. 

Comparing these findings with those provided by enrichment analysis (Table 3) four genes in particular, EGFR, MAPK3, MTOR, and PIK3R1, were identified as the main anti-liver cancer targets of *F. indica* and were chosen for molecular docking experiments. 

### 2.4. GO and KEGG Pathway Analysis

**Figure 2:**
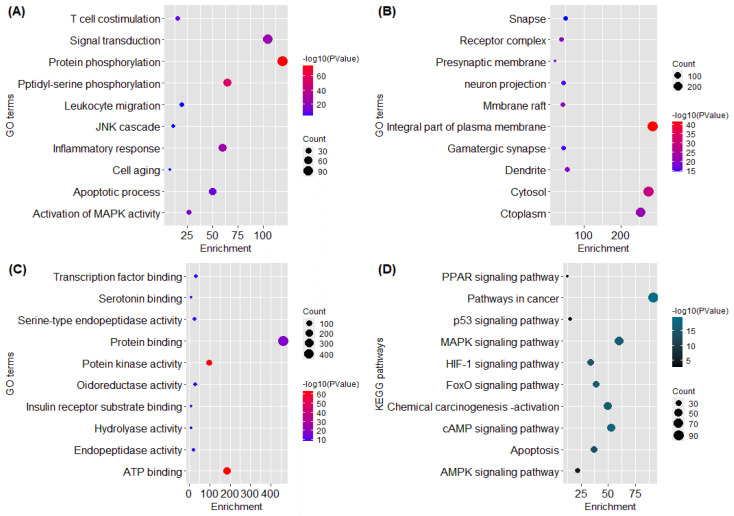
Representation of functional annotation and enriched pathways in form of Bubble Plot. (**A**) GO in terms of biological processes. (**B**) GO in terms of molecular function. (**C**) GO in terms of cellular components. (**D**) KEGG pathway analysis.

**Figure 3:**
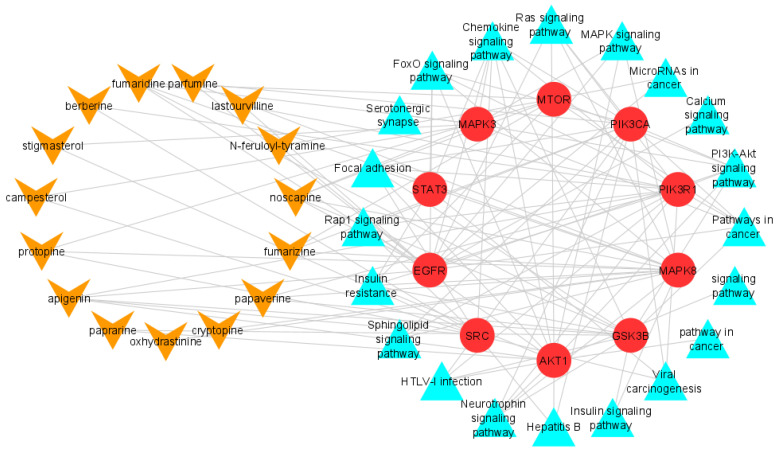
Pathways influenced by *F. indica*. The red nodes represent the hub genes, the orange nodes represent active compounds, and the blue nodes are the pathways associated with the core targets.

The functional annotation and enrichment analysis unveiled the potential biological roles of *F. indica* targets. The targets of *F. indica*, according to GO functional analysis, were related to protein phosphorylation, inflammatory response, integral part of plasma membrane, and so on. (Figure 2). The KEGG pathway analysis was performed to identify the significant signaling pathways linked to the anti-liver cancer effect of *F. indica*. It is noteworthy that most of the genes were involved in following pathways. These include the NF-κB, the stress-responsive mitogen-activated protein kinase (MAPK), and the STAT pathways [19], including the PI3K-AKT-mTOR, AMPK-mTOR, EGF, MAPK, Wnt/β-catenin, p53, and NF-κB pathways [20]. KEGG pathway analysis revealed that EGFR, MAPK3, MTOR, and PIK3R1 were significantly enriched genes (Figure 3).

### 2.5. Molecular Docking

**Figure 4:**
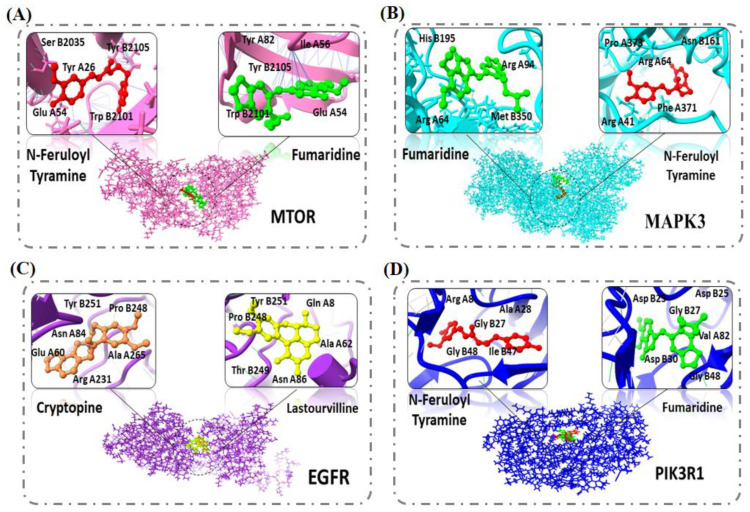
Represents the sketch maps of target proteins together with their strongest binding components. (**A**) MTOR, (**B**) MAPK3, (**C**) EGFR, and (**D**) PIK3R1.

The top four targets EGFR, MTOR, MAPK3, and PIK3R1 were chosen for molecular docking after a thorough analysis of the PPI network. PDB structure was used to find the 3D structure of the target protein (EGFR (PDB id: 1IVO), MTOR (PDB id: 1FAP), MAPK3 (PDB id: 2ZOQ), and PIK3R1 (PDB id: 1HPO)). All the PDB structures were selected on the basis of their resolution. Low numeric values in Å mean the resolution of the structure is good and can be considered for further analysis. In the framework of the current study, EGFR had a resolution of 3.30 Å, while other targets MTOR, MAPK3, and PIK3R1 had resolution of 2.70 Å, 2.39 Å, and 2.50 Å, respectively. Furthermore, the CPort tool was used for active site prediction of the selected proteins. The compounds interacted with the active site of the EGFR receptor via forming a bond with the following amino acid residues: Tyr B251, Gln A8, Leu A38, Ala A62, Asn A86, Thr B249, Pro B248, and Lys A407. In the case of MTOR, the active compounds bound with the Ser B2035, Glu A54, Tyr A26, Phe A46, Asp A37, Arg A42, Thr B2098, Asp B2102, Lys B2095, Trp B2101, Phe B2039, Tyr B2105, Phe B2108, and Leu B2031 residues. On the other hand, MAPK3 bound with active compounds via Arg A41, Arg A64, Thr B347, Glu B194, Pro B193, Asn B161, Phe A371, Arg A370, Pro A373, and Asp A105. Lastly, the selected compounds bound with PIK3R1 by forming a bond with Arg A8,Ala A28,Gly B27,Asp A25,Ile A84,Asp B25,Gly B48,Ile B47,Asp B29,Ile A50,Gly A4, and Ile B50 (Figure 4). The drug candidates showed hydrogen bond, Pi–pi-stacked, and van der Waals interactions with the receptor proteins, indicated with dotted lines mentioned in the additional file (Supplementary Material: Table S1). All those binding pockets were selected by the site finder tool present in the molecular operating environment. The top four inhibitors, Fumaridine, N-feruloyl tyramine, Cryptopine, and Lastourvilline, were screened out of the 15 showing a good docking score along with RMSD values for all targets as shown in Table 4. 

Fumaridine and N-feruloyl tyramine showed good binding affinity with all the targets, except EGFR, having docking scores between −13.86 kcal/mol and −10.69 kcal/mol. However, EGFR showed top binding with the compounds Lastourvilline and Cryptopine, having docking scores of −12.69 kcal/mol and −10.29 kcal/mol, respectively. Similarly, all the compounds also showed strong hydrogen bond interactions with interacting residues of MTOR. Thus, docking analysis strengthened our findings that predicted stable target bonds with active compounds of *F. indica*. Figure 4 represents the sketch map of target proteins together with their strongest binding components.

### 2.6. ADMET Profiling

ADMET analysis is a challenging process in drug discovery. The SwissADME tool was applied to forecast different types of pharmacokinetic properties. The pharmacokinetic factor may be used to predict the absorption, distribution, metabolism and elimination (ADME), and toxicity of the top therapeutic novel compounds. ADMET profiling of all the top selected drug candidates showed that there are no negative consequences of the pharmacokinetic properties, first and foremost, of the potential compounds (Table 5). The ADMET characteristics of possible drugs for various models such as P-glycoprotein substrates, BBB penetration, and CYP2C19 inhibitors, CYP2C9 inhibitors, CYP2D6 inhibitors, and CYP3A4 inhibitors produced promising results that substantially confirm the compound ability to function as a drug candidate. Furthermore, the skin permeation lop Kp values describe that, depending on its size and chemophysical qualities, a chemical can permeate the stratum corneum via intercellular, transcellular, or appendageal channels. It is noteworthy that all the compounds showed non-toxic behavior, although different types of toxicity were measured for all compounds, and none of the compounds showed toxic behavior.

## 3. Discussion

Natural product research has received a lot of interest in recent years [21]. The network pharmacology method aids in the understanding of the complicated interactions that exist between medicines and their targets, as well as the probable mechanisms of action [22,23]. Moreover, the diversity of developing new drugs from plant sources provides methodological challenges [24]. Because of the lack of ADME qualities in the newly discovered medication, and because of the high-budget nature of research, drug discovery methodologies face additional challenges [25]. As a result, in the creation of medications, pharmaceutical specialists place a high value on ADME-based screening [26]. Liver fibrosis, viral hepatitis, fatty liver, cirrhosis, and liver cancer are all serious disorders that endanger human health and are the top causes of mortality globally [27]. Despite significant advances in the treatment of liver disorders over the last several centuries, the majority of medications still fail to provide satisfying results in patients [28]. Hepatocellular carcinoma is significantly linked to chronic hepatitis B virus (HBV) or hepatitis C virus (HCV) infection, aflatoxin-contaminated food consumption, and high alcohol usage [29]. Multiple drug resistance (MDR), a high clearance rate, severe side effects, undesirable drug distribution to the specific site of liver cancer, and a low concentration of medication that reaches liver cancer cells are just a few of the drawbacks of traditional liver cancer chemotherapy. As a result, new techniques and network pharmacology must be developed to convey the medication molecules specific to the malignant hepatocytes in enough of an amount and for a sufficient period of time within the therapeutic window [30,31]. As a consequence, the search for novel drugs is becoming targeted. A high-potency origin of phytochemicals with medical advantage would have a potential liver cancer therapy option in this scenario. 

*Fumaria indica* is a medicinal plant of the fumitory family that is rich with phytochemical constituents, which have huge medicinal value [32,33]. *F. indica* has antipyretic, antidiarrheal, and hypoglycemic effects, according to pharmacological investigations [34,35]. Various in vitro studies revealed the therapeutic significance of *F. indica* against liver diseases. However, the exact molecular mechanism remains unclarified [8]. This study provides a foundation for the initial screening of *F. indica* bioactive compounds, as well as a novel therapeutic concept for future research into *F. indica* processes for liver cancer therapy. The hallmark of this age will be the identification of potential bioactive ingredients that stop the pathophysiology of disorders and disease. 

In the current study we uncover several target genes that are revealed to be involved in various pathways in cancer. The pathogenesis of disease can be avoided by targeting the genes that cause disruption in pathways in cancer. A slew of studies strengthened our findings, such as most people that are suffering from liver cancer include chronic infections with HBV or HCV, or cirrhosis, and certain people inherit liver diseases and diabetes [36]. It is important to note that two of our major targets, MTOR and MAPK3, are primarily implicated in liver cancer resistance pathways [37]. Our research proposed that MTOR, MAPK3, and EGFR are directly involved in hepatitis B pathways. As a result, changes in these genes may disrupt the pathways that interconnect them, leading to disorder. Beyond this, the targeted genes of active constituents are also enriched in various inflammatory conditions such as arthritis and so forth, which seems to indicate that they can act on various antiinflammatory cytokines and exert an effect on liver cancer. It is worth noting that our key gene, namely EGFR, has been shown to play a key role during liver regeneration following acute and chronic liver damage, as well as in cirrhosis and hepatocellular carcinoma, highlighting the importance of EGFR in the development of liver diseases [38]. Hence blocking the EGFR genes might help in the treatment of liver cancer. Furthermore, the mammalian target of the rapamycin (mTOR) signaling system is involved in many aspects of cancer such as cell growth, the inhibition of apoptosis, and metabolic reprogramming proliferation [39]. This demonstrates conclusively that the dysregulation of mTOR is emphasized in the pathogenesis of liver cancer.

The biological information of target genes was obtained using GO enrichment analysis. According to GO functional analysis, anti-liver cancer targets of *F. indica* were mainly involved in protein phosphorylation, peptidyl-tyrosine phosphorylation phosphatidylinositol 3-kinase complex, class IA, and GO protein serine/threonine/tyrosine kinase activity. KEGG pathway studies revealed that targets were involved in liver cancer-related pathways. The KEGG pathway enrichment results revealed that the putative targets were significantly enriched in hepatitis B and viral carcinogenesis, and the cAMP signaling pathway, PI3K-Akt signaling route, MAPK signaling pathway, estrogen signaling pathway, p53 signaling pathway, and cell cycle signaling pathway were all found to be enriched in cancer pathways. 

It is noteworthy that our core genes are mainly enriched in the cyclic adenosine monophosphate (cAMP) signaling pathway. Previous studies demonstrated that the cAMP signaling pathway controls a variety of cellular activities such as lipid, metabolism, inflammation, cell differentiation, and injury and regulates gene-protein expression and function [40]. Hence, disturbance in the cAMP signaling pathway might be associated with liver cancer. Furthermore, through the KEGG pathways it has been revealed that MAPK3 genes are directly involved in the mitogen-activated protein kinase (MAPK) signaling pathway. MAPK inhibitors are effective at reducing pro-inflammatory cytokinesis and increasing anticancer activity, especially in human pancreatic cancer cells [31]. This gives clear evidence that dysregulation of the MAPK3 gene causes disturbance in the MAPK signaling pathway, which ultimately leads to liver cancer. Therefore, by targeting MAPK3, the pathophysiology of liver cancer can be halted. Beyond this, the targeted genes of active constituents are also enriched in various other cancer-related signaling pathways, which seems to indicate that these compounds and their associated target genes exert a strong effect on liver cancer.

According to topological parameters of the compound–genes–pathway network, four major targets named EGFR, MAPK3, MTOR, and PIK3R1 were identified as the core targets. Furthermore, these core targets were validated using molecular docking, which revealed that Fumaridine, N-feruloyl tyramine, Lastourvilline, and Cryptopine bound stably with these core targets. The findings of docking analysis indicated that these four compounds can be used for treatment of liver cancer because of their ability to bind stably with core targets. In the light of current network pharmacology, this research predicted the active compounds, their prospective targets, and associated pathways for the treatment of liver cancer, thereby providing a theoretical foundation for future experimental research. Given the limitation of network pharmacology, the basic pharmacological mechanism for liver cancer treatment can only be discovered by data mining. The mining of active compounds is particularly based on different databases. Although the information in databases are curated, a lot of inconsistencies may, however, occur due to variety of information resources and experimental data. In this regard, modern high throughput techniques including chromatography can be used; liquid chromatography and mass spectrometry can help to solve this problem. Even though we have given some intriguing evidence, more research and clinical trials are required to fully investigate the potential of *F. indica* and to validate its medicinal applications.

## 4. Materials and Methods

### 4.1. Virtual Screening of Active Constituents

The information on active phytocompounds of *Fumaria indica* was collected from the literature using different databases such as PubMed and Google scholar and KNApSAcK. PubChem Explore Chemistry [41] was used to obtain the Canonical Simplified Molecular-Input Line-Entry System (SMILES) of each active ingredient, while PubMed [42] and ChemSpider [43] were used to obtain the chemical structures of active compounds. All constituents of *F. indica* were virtually screened by applying bioavailability (OB) and drug likeness (DL) parameters, which are crucial in the characteristics of absorption, distribution, metabolism, and excretion (ADME) characteristics of drugs. Compounds were only retained if DL ≥ 0.18 and OB ≥ 30% to satisfy ADME criteria. Biologically active compounds that did not match these conditions were discarded and were not investigated further. In this regard, DL and OB of all active constituents were calculated using Molsoft [44] and SwissADME [45].

### 4.2. Target Genes Screening

The potential target genes of screened active constituents were retrieved by entering their Canonical SMILES to SwissTarget Prediction tool [46]. Therefore, the target with probability ≥ 0.7 were selected. Prediction of disease-related genes is the next step to uncover the molecular mechanism of medicinal herb to treat multiple diseases. Two databases, GeneCards and DisGeNET, were searched using keywords ‘primary liver cancer’ and ‘Hepatic cancer’ to retrieve disease-related genes. DisGeNET is a multipurpose data system that provides information related to genes, disorders, and their related empirical studies [47]. GeneCards database contains information related to the genome, proteome, and transcriptomes of an organism [48]. The Venn online tool was used to identify the overlap genes between predicted target genes of screened compounds and disease-related targets [49]. Therefore, the common targets of active constituents and disease were obtained for subsequent analysis.

### 4.3. Pathway and Functional Enrichment Analysis

To perform gene enrichment analysis and KEGG pathway analysis, Database for annotation, visualization, and integrated discovery (DAVID) [50] was used. List of key genes were subjected to DAVID to perform functional annotation at three levels: cellular component (CC), molecular function (MF), and biological process (BP). DAVID is a web-based functional enrichment database that helps researchers to comprehend the bioactivity of a huge number of genes. In current study, *p*-value ≤ 0.01 was selected, and top 10 GO enrichments and top 10 KEGG pathways were chosen for subsequent analysis.

### 4.4. Network Construction

The mechanism of *F. indica* in liver cancer was performed by network analysis. The software Cytoscape 3.8.0, which is a freely available, graphical user interface for importing, visually exploring, and analyzing bimolecular interaction networks, was used to construct and visualize the network [51]. Active constituents and the target genes in the network were represented by nodes, while the edges were used to represent the interaction between active constituents and their target genes. Network analyzer tool was used to calculate degree, a topological property that reveals the importance of compound-target gene-pathways in network diagram. Moreover, target genes with the highest degree of connectivity were considered as ‘key target’.

### 4.5. PPI Network Construction and Molecular Docking Analysis

**Figure 5:**
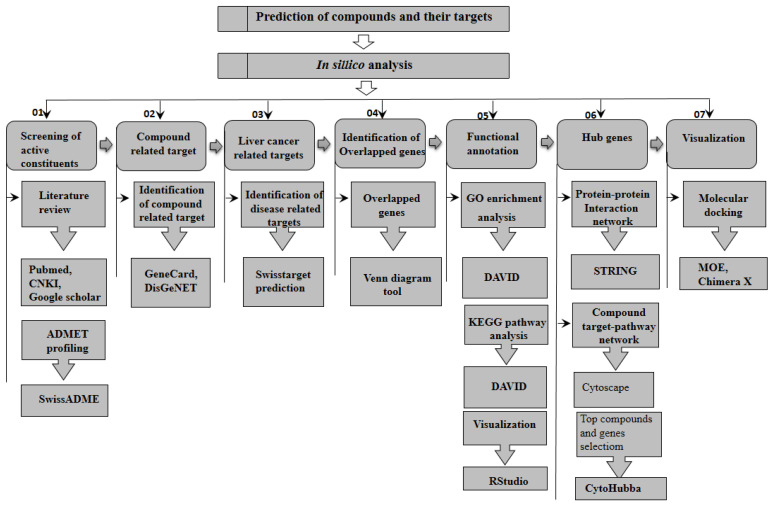
Graphical synopsis representing the overall strategy used in the prediction of potential compounds and their potential targets for liver cancer treatment.

Protein–protein interaction (PPI) data were obtained from the Search Tool for the retrieval of Interacting Genes (STRING) database with a confidence score of >0.7 to construct PPI network by uploading common genes on a database [52]. The PPI network obtained from STRING was subjected to the cytoHubba plugin of Cytoscape, which was used to analyze the core regulatory genes of the PPI web and the identification of key targets. The observed co-expression of predicted key targets was also obtained through STRING database. Moreover, key targets were validated through molecular docking approach. The RCSB PDB [53] was used to obtain the X-ray crystal structure of candidate target; it was used to obtain crystal structures of potential targets. Moreover, refinement of structure was performed using Chimera. After that, they were brought into molecular operating environment (MOE) [54], which was used to extract ligands from proteins, adjust their structure, and remove water from them. The best docked score with the lowest RMSD and binding energy were selected for further analysis. Furthermore, Chimera X and discovery studio was used for visualization of interaction among active compounds and predicted target. The workflow of the present study is displayed in Figure 5.

## 5. Conclusions

This research establishes a scientific foundation for determining the efficacy of multicomponent, multi-target drug treatment as well as finding novel anti liver cancer therapeutic targets. In this study, network pharmacology along with molecular docking was employed to explore the underlying mechanism for the treatment of liver cancer. According to network analysis, *F. indica* contains multi-targeting compounds that function on numerous disease-related pathways; hence, they might be considered as novel therapeutic options against liver cancer. Furthermore, our studies revealed that the EGFR, MAPK3, MTOR, and PIK3R1 genes are effective and potential therapeutic agents for lowering the incidence of liver cancer and potentially exhibiting therapeutic effects on liver cancer. However, the current study has significant limitations since further phytochemical and pharmacological research is needed to verify these findings.

## Figures and Tables

**Table 1:** Fifteen active compounds, their properties and structures.

Molecule Name	Molecular Weight (MW)	Drug Likeness (DL)	Oral Bioavailability (OB)	Structure	PubChem ID
Protopine	353.37	0.29	0.55	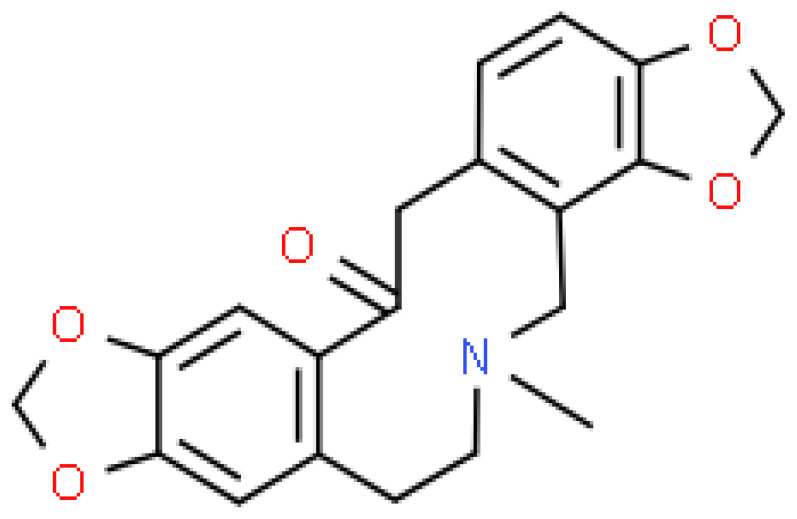	4970
Fumaridine	396.44	0.55	0.55	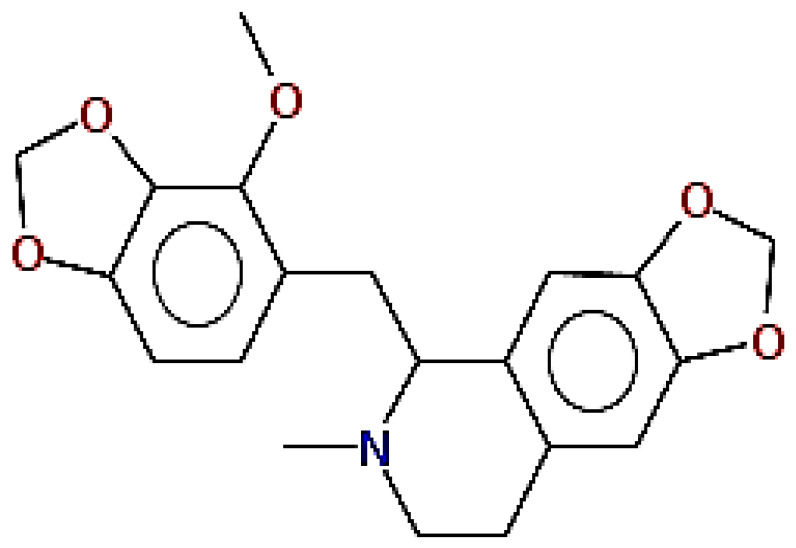	6537302
Parfumine	353.37	0.33	0.55	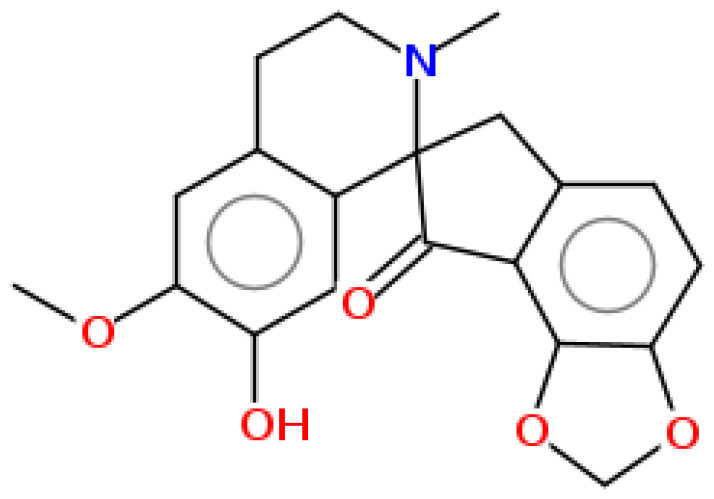	185623
Lastourvilline	327.37	0.74	0.55	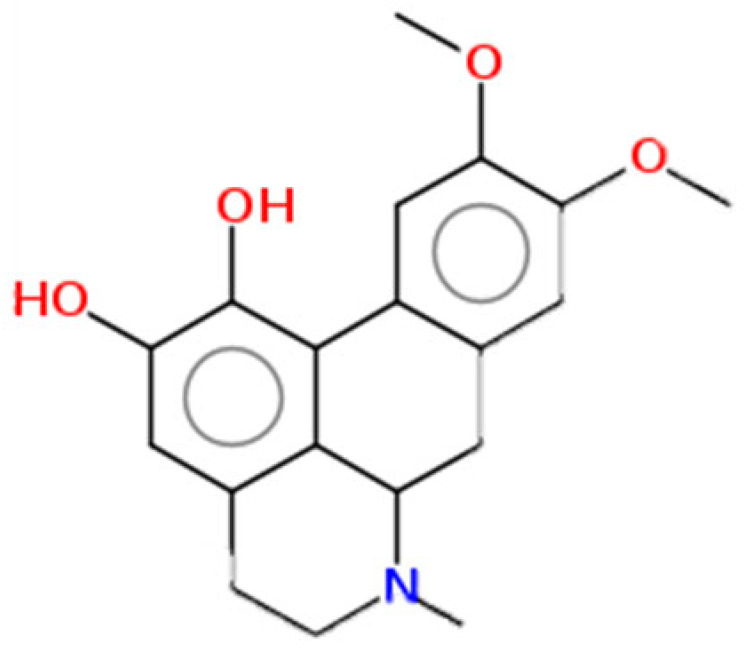	155514
N-feruloyl tyramine	313.35	0.21	0.55	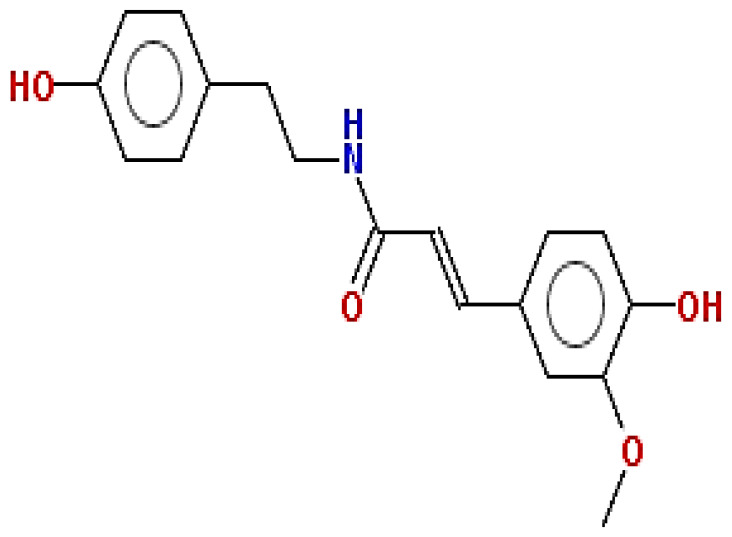	45257345
Fumarizine	355.38	1.06	0.55	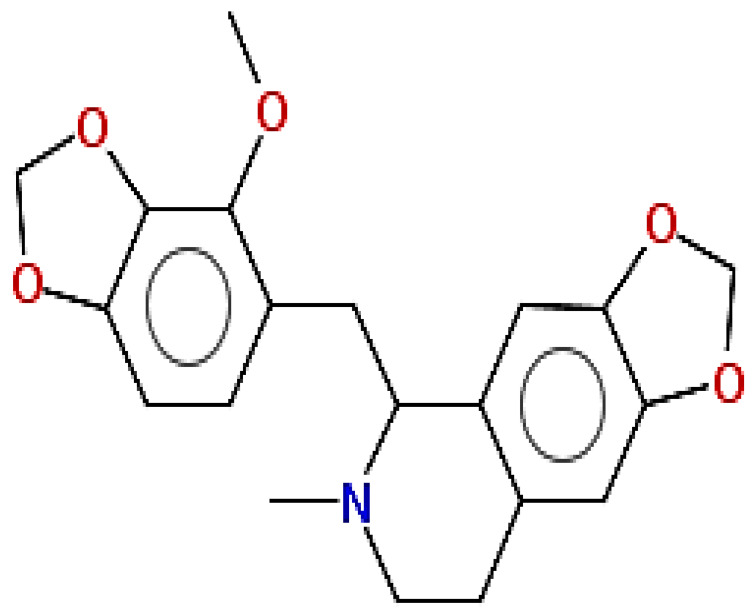	11131999
Paprarine	397.38	0.3	0.55	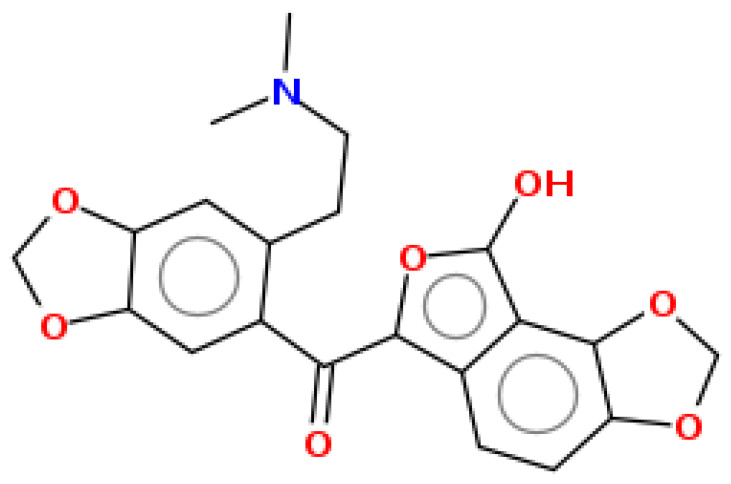	15226320
Cryptopine	369.41	0.35	0.55	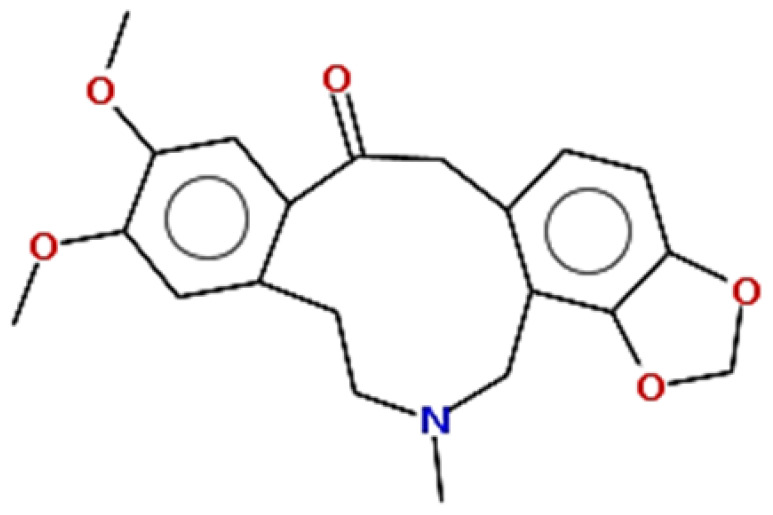	72616
Berberine	336.36	0.77	0.55	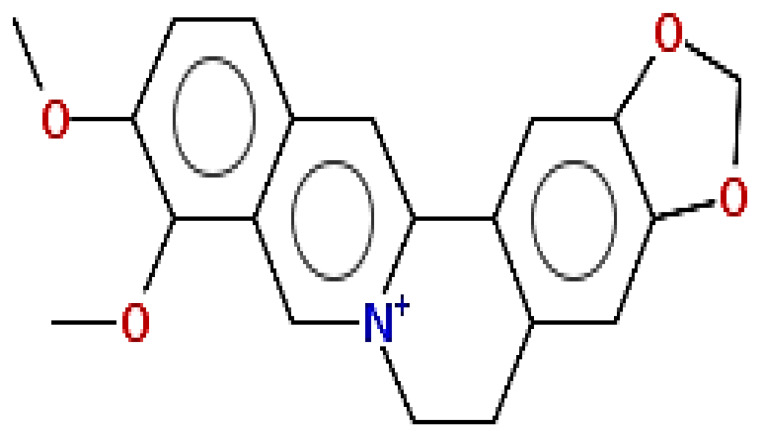	2353
Stigmesterol	412.69	0.62	0.55	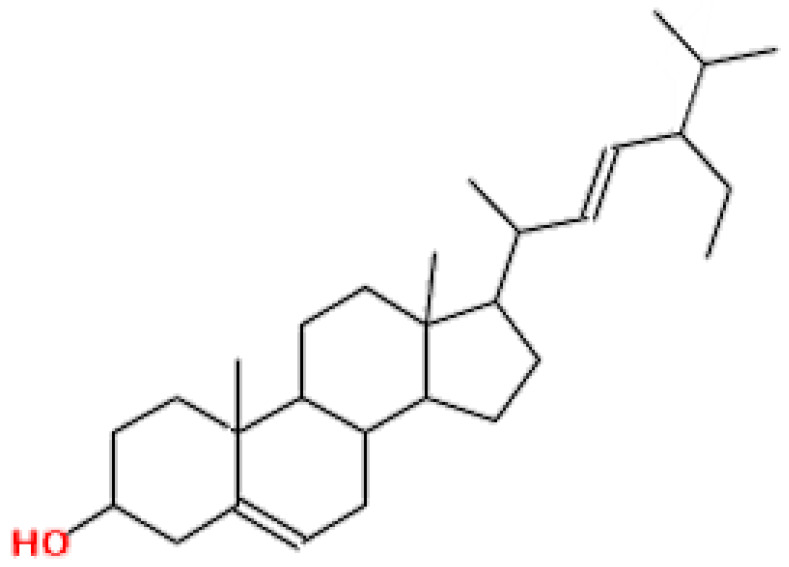	5280794
Campesterol	400.68	0.59	0.55	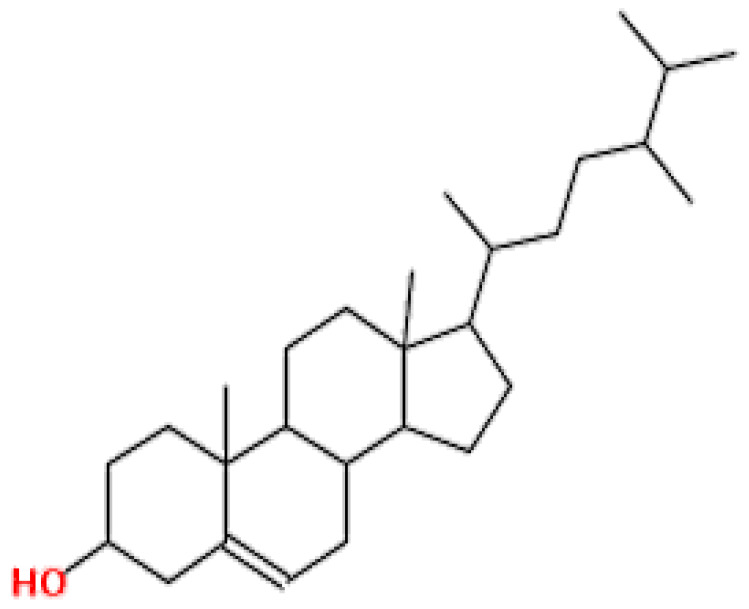	173183
Papaverine	339.39	0.75	0.55	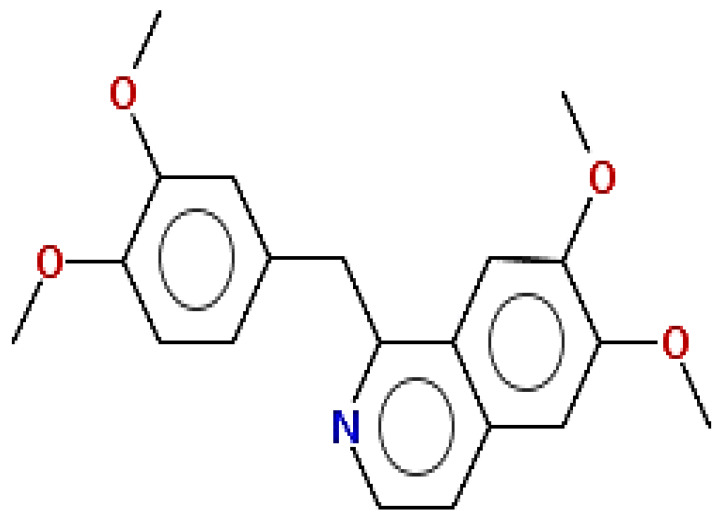	4680
Oxyhydrastinine	205.21	0.18	0.55	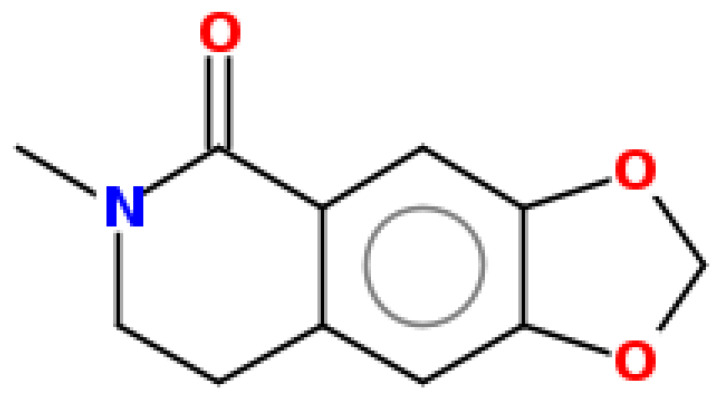	160522
Noscapine	413.42	0.54	0.55	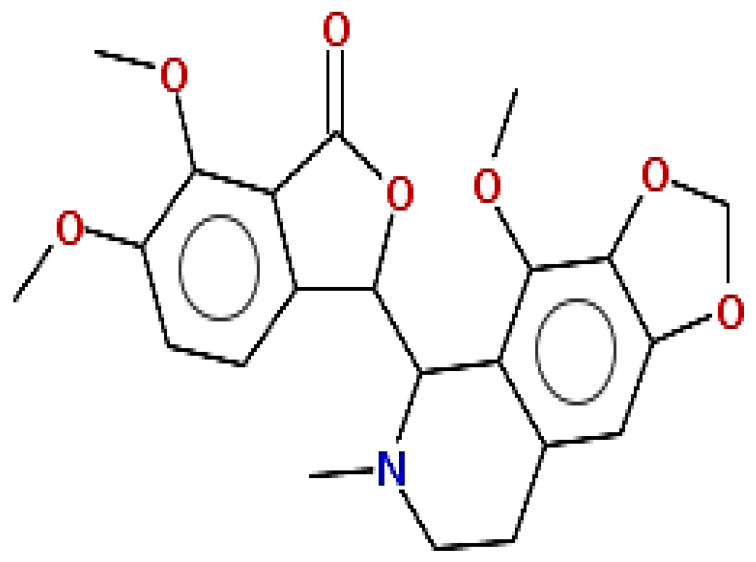	275196
Apigenin	270.24	0.39	0.55	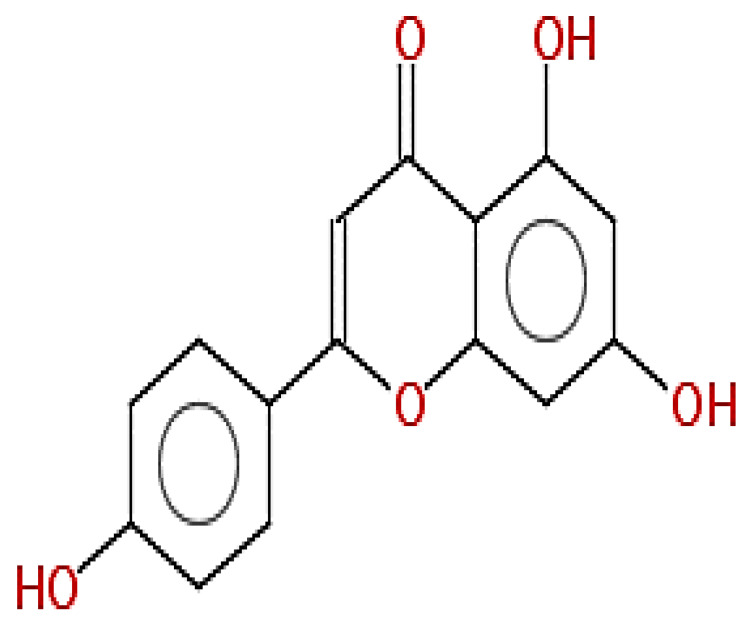	5280443

**Table 2:** Degree of 12 compounds explored through network analyzer in Cytoscape.

Molecule Name	Class	Degree
Protopine	Alkaloids	4
Fumaridine	Alkaloids	8
Parfumine	Alkaloids	3
Lastourvilline	Alkylamides	1
N-Feruloyltyramine	Tyramines	4
Cryptopine	Alkaloids	7
Berberine	Alkaloids	3
Stigmasterol	Steroid	3
Campesterol	Steroid	2
Papaverine	Alkaloids	2
Oxhydrastinine	Alkaloids	2
Noscapine	Alkaloids	6

**Table 3:** Top 10 genes ranked by degree method.

Gene Name	Compounds	Score	Pathways
AKT1	Fumaridine/Paprarine/Apigenin	224	Neuroactive ligand–receptor interaction, pathways in cancer, cAMP signaling pathway,
TNF	N-feruloyl tyramine	207	Proteoglycans in cancer, MAPK signaling pathway, insulin resistance
SRC	Protopine/Stigmasterol/Fumaridine/Berberine Campesterol/Cryptopine/Apigenin	184	Chemokine signaling pathway, viral carcinogenesis
EGFR	Fumaridine/Parfumine/Lastourvilline/N-feruloyl tyramine/Noscapine/Apigenin	169	Focal adhesion, Rap1 signaling pathway, serotonergic synapse
STAT3	Cryptopine	167	Pathway in cancer, proteoglycans in cancer, FoxO signaling pathway
MAPK3	Fumaridine/Cryptopine/Stigmasterol/Campesterol/, Noscapine	165	Viral carcinogenesis, focal adhesion, Rap1 signaling pathway
CASP3	Oxyhydrastinine	155	Pathways in cancer, proteoglycans in cancer, MAPK signaling pathway, Hepatitis B
MTOR	Protopine/Fumaridine/N-feruloyl tyramine/Fumarizine, Cryptopine/ Noscapine	135	MicroRNAS in cancer, insulin resistance
MAPK1	Protopine/Fumaridine/Cryptopine/Noscapine	134	Neurotrophin signaling pathway, serotonergic synapse
PIK3R1	Apigenin/Lastourvilline/Cryptopine/Berberine/Papaverine	94	Sphingolipid signaling pathway, Hepatitis B

**Table 4:** Binding energy and interactions of potential active compounds and their four target proteins.

MTOR				
Compound ID	Compound Name	Docking Score (kcal/mol)	RMSD	Hydrogen Bond and Other Interacting Residues
6537302	Fumaridine	−13.86	1.71	Tyr A82, Tyr B2105, Phe B2108, Phe B2039, Ile A56, Phe A46, Glu A54, Trp B2101
5280537	N-feruloyl tyramine	−12.94	0.93	Ser B2035, Glu A52, Tyr A26, Phe A46, Asp A37, Arg A42, Thr B2098, Asp B2102, Lys B2095, Trp B2101, Phe B2039, Tyr B2105, Phe B2108, Leu B2031
72616	Cryptopine	−10.95	1.4349	Phe B2039, Ile A56, Tyr A82, Thr B2098, Arg A42, Asp A37, Phe A46, Asp B2102
155514	Lastourvilline	−9.77	2.9765	Phe B2039, Tyr A82, Glu A54, Ser B2035, Trp B2101, Ser B203
**Standard Drug**				
54675783	Minocycline	−7.79	0.79	ASP A37 PHE B2039
**MAPK3**				
6537302	Fumaridine	−12.32	1.17	His B195, Arg A64 Met B350, Arg A94
5280537	N-feruloyl tyramine	−12.07	1.77	Asn B161, Phe A371, Arg A64, Pro A373, Arg A41, Thr B347, Glu B194, Arg A104
72616	Cryptopine	−10.1110	1.01	Arg A64, Arg A41, Arg A104, Asp A105, Phe A371, Pro B193, Asn B161, Asp B192, IIe B190
155514	Lastourvilline	−11.0807	1.31	Arg A41, Arg A64, Thr B347, Glu B194, Pro B193, Asn B161, Phe A371, Arg A370, Pro A373, Asp A105
**Standard Drug**				
54675783	Minocycline	−7.75	2.9235	Arq A41,Asp B192
**EGFR**				
155514	Lastourvilline	−12.6598	1.8080	Tyr B251, Gln A8, Leu A38, Ala A62, Asn A86, Thr B249, Pro B248, Lys A407
72616	Cryptopine	−10.2961	0.7945	Tyr B251, Arg A84 Ala A62, Thr B249, Pro B248, Asn A86, Glu A60, Arg A231, Ala A265, Leu A38, Gly A264
6537302	Fumaridine	−10.12	1.5566	Tyr B251, Lys A322, Asn A86, Thr B249, Ala A62, Pro B248, Leu A38, Lys A407, Met A87
5280537	N-feruloyl tyramine	−10.27	2.4809	Asn A12, His A409, Ser A11, Gly A410, Thr A10, Arg A285, Lys A407, Arg A405, Tyr B251, Leo A38, Gln A8, Gly C39
**Standard Drug**				
176870	Erlotinib	−8.06	2.3660	Arg A231
**PIK3R1**				
6537302	Fumaridine	−12.10	3.0608	Agr A8, Asp B30, Val A82, Ile A84, Asp B25, Gly B27, Gly B48, Ala B28, Asp B29, Asp B30
5280537	N-feruloyl tyramine	−10.69	3.4899	Arg A8, Ala A28 Gly B27, Asp A25 Ile A84, Asp B25, Gly B48, Ile B47, Asp B29, Ile A50 Gly A49, Ile B50
72616	Cryptopine	−10.45	1.2321	Arg A8, Ala A28, Asp A25, Asp B25, Ala B28, Gly B27, Leu A23, Arg A8, Val A82, Gly B48, Pro A81, Ile B50, Gly A49
155514	Lastourvilline	−10.02	0.7393	Asp B30, Ala B28, Arg A8, Asp A25, Pro A31, Ile B50, Val A32, Gly B49, Gly B48, Asp B29
**Standard Drug**				
49867926	XL-765	−9.80	1.73	Asp B25

**Table 5:** ADMET profiling of compounds.

Standard Parameters	Fumaridine	N-Feruloyl Tyramine	Cryptopine	Lastourvilline
GI absorption	High	High	High	High
BBB	Yes	No	Yes	Yes
P-gp substrate	Yes	No	Yes	Yes
CYP1A2 inhibitor	No	No	Yes	Yes
CYP2C19 inhibitors	Yes	No	No	No
CYP2C9 inhibitors	Yes	No	Yes	No
CYP2D6 inhibitors	Yes	Yes	Yes	Yes
CYP3A4 inhibitors	Yes	Yes	Yes	Yes
Log Kp (skin permeation)	−6.62 cm/s	−6.72 cm/s	−6.48 cm/s	−6.71 cm/s
**Toxicity**				
Carcinogens	Non-carcinogenic	Non-carcinogenic	Non-carcinogenic	Non-carcinogenic
Cytotoxicity	Non-toxic	Non-toxic	Non-toxic	Non-toxic
Mutagenicity	Nil	Nil	Nil	Nil

## Data Availability

Data is contained within the article and Supplementary Material.

## Supplementary Materials

The following supporting information can be downloaded at: https://www.mdpi.com/article/10.3390/ph15060654/s1. Table S1: Interaction analysis of docked complexes.
