# Supplementary material for: Network Pharmacology and Bioinformatics Approach Reveals the Multi-Target Pharmacological Mechanism of Fumaria indica in the Treatment of Liver Cancer
Source: Pharmaceuticals (Basel). 2022 May 25;15(6):654. doi: 10.3390/ph15060654 (PMC9229061; doi:10.3390/ph15060654)
Supplement: Supplementary file 1 [file pharmaceuticals-15-00654-s001.zip › pharmaceuticals-1707361-supplementary.pdf]

Table S1: Interaction analysis of docked complexes

<table>
<tr><th colspan="2">MTOR</th></tr>
<tr><td>Fumaridine

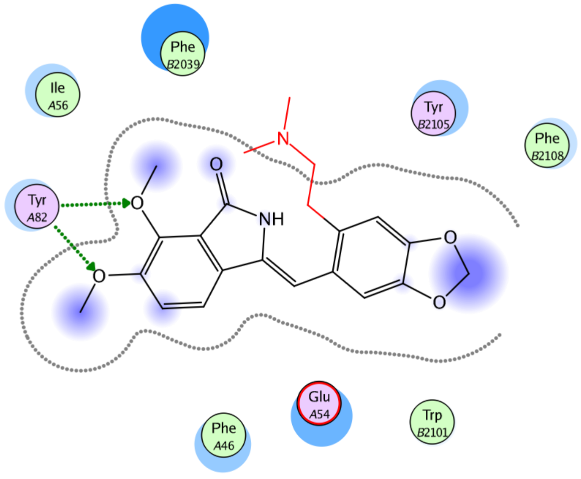
</td>
<td>N-feruloyl tyramine

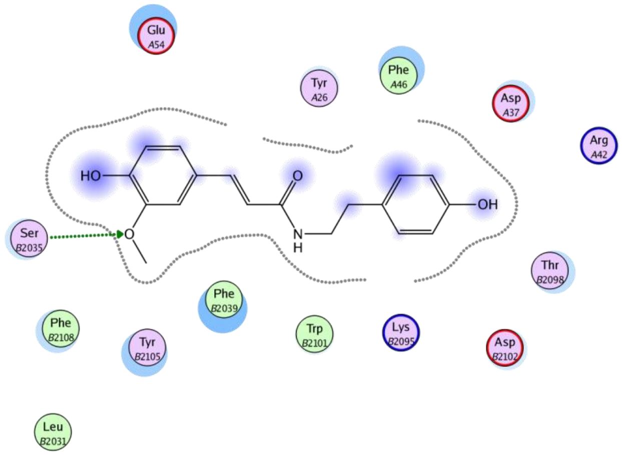
</td></tr>
<tr><td>Cryptopine

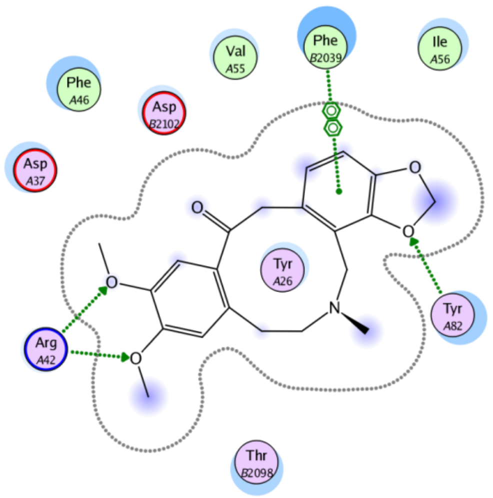
</td>
<td>Lastourvilline

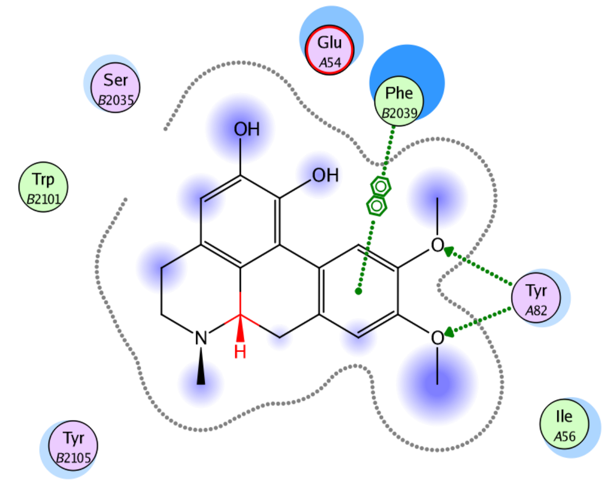
</td></tr>
<tr><th colspan="2">MAPK3</th></tr>
<tr><td>Fumaridine</td><td>N-feruloyl tyramine</td></tr>
</table>

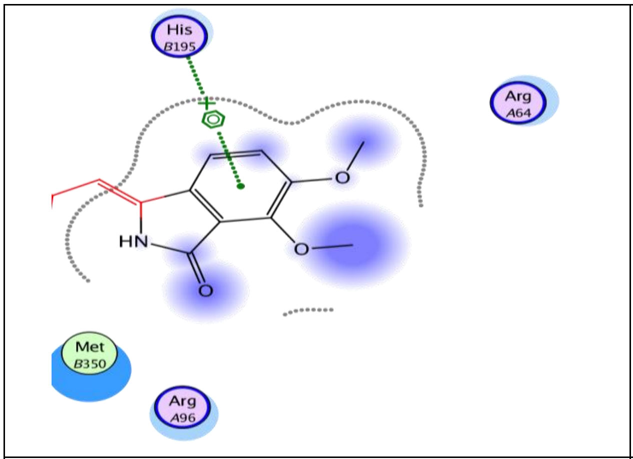

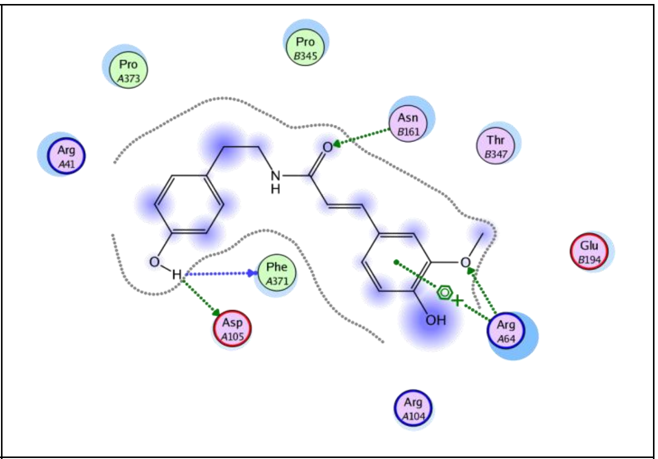

Cryptopine

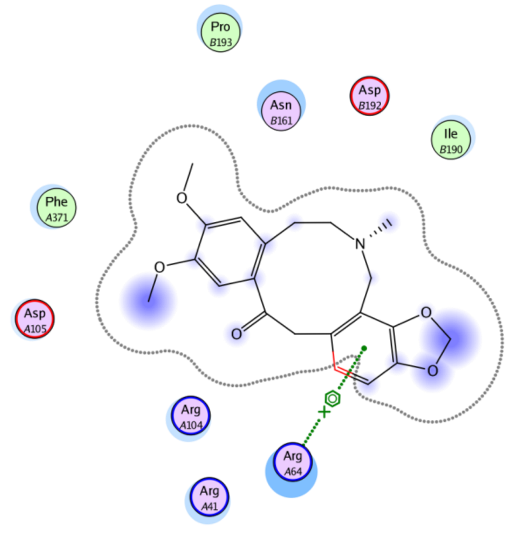

Lastourvilline

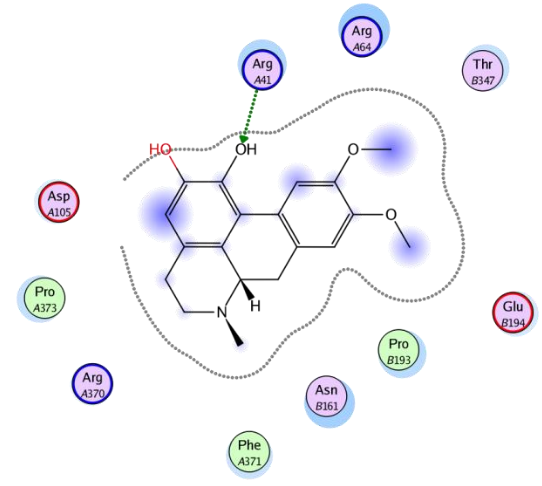

EGFR

Lastourvilline

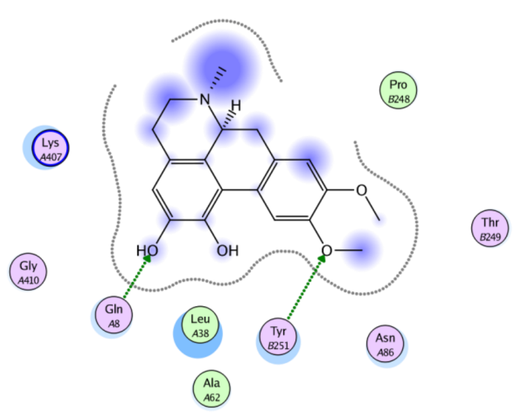

Cryptopine

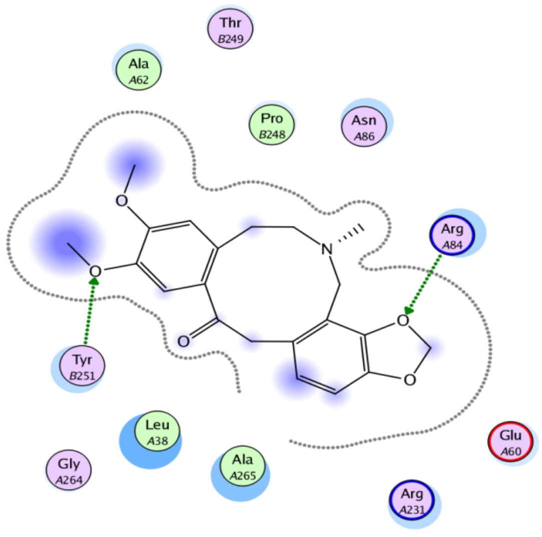

Fumaridine	N-feruloyl tyramine
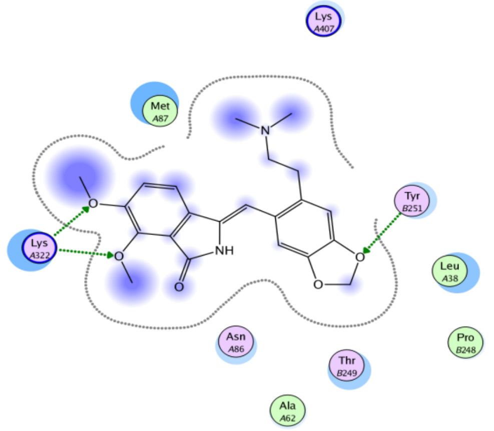	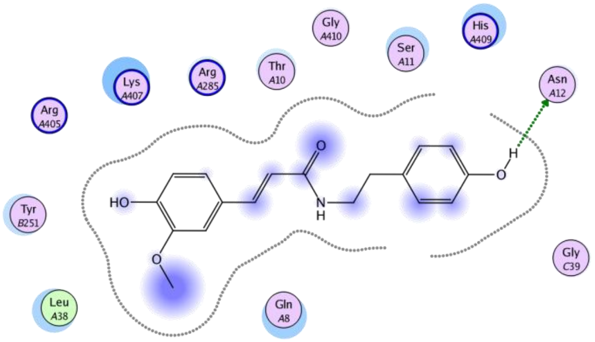

PIK3R1

Fumaridine	N-feruloyl tyramine
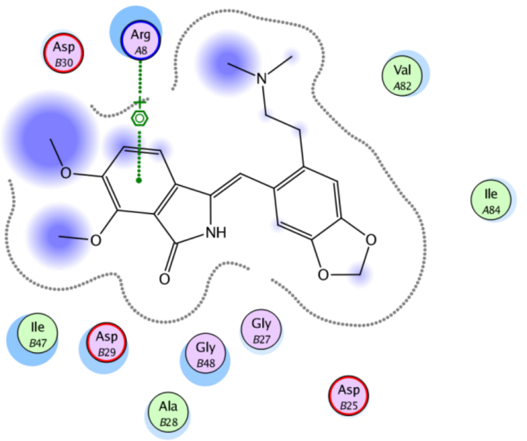	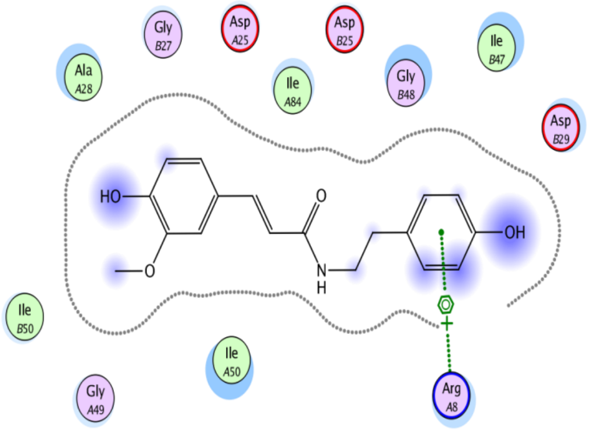

Cryptopine	Lastourvilline
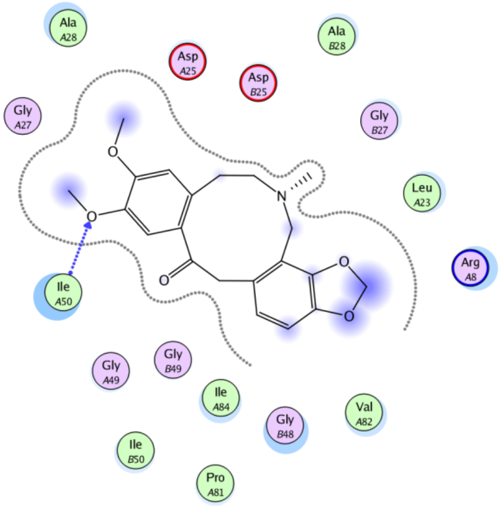	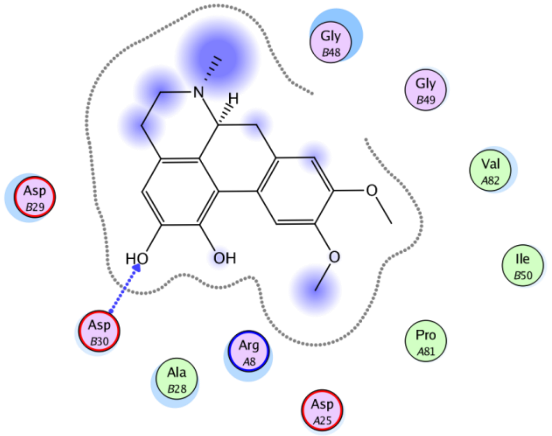
